# The Creativity Diamond—A Framework to Aid Creativity

**DOI:** 10.3390/jintelligence10040073

**Published:** 2022-09-22

**Authors:** Peter Childs, Ji Han, Liuqing Chen, Pingfei Jiang, Pan Wang, Dongmyung Park, Yuan Yin, Elena Dieckmann, Ignacio Vilanova

**Affiliations:** 1 Dyson School of Design Engineering, Imperial College London, London SW7 2AZ, UK; 2 Department of Management, Business School, University of Exeter, Exeter EX4 4PU, UK; 3 College of Computer Science and Technology, Zhejiang University, Hangzhou 310027, China; 4 School of Engineering and the Environment, Kingston University, London SW15 3DW, UK; 5 School of Design, Hong Kong Polytechnic University, Hong Kong, China; 6 Division of Design, Incheon National University, Incheon 22012, Korea

**Keywords:** creativity, creativity process, creativity tools, thinking, idea generation and evaluation

## Abstract

There are many facets to creativity, and the topic has a profound impact on society. Substantial and sustained study on creativity has been undertaken, and much is now known about the fundamentals and how creativity can be augmented. To draw these elements together, a framework was developed called the creativity diamond, formulated on the basis of reviews of prior work, as well as the consideration of 20 PhD studies on the topics of creativity, design, innovation, and product development. The framework embodies the principles that quantity of ideas breeds quality through selection, and that a range of creativity tools can provoke additional ideas to augment our innate creativity. The creativity diamond proposed is a tool consisting of a divergent phase associated with the development of many distinctive ideas and a convergent phase associated with the refinement of ideas. The creativity diamond framework can be used to prompt and help select which tool or approach to use in a creative environment for innovative tasks. The framework has now been used by many students and professionals in diverse contexts.

## 1. Introduction

The term creativity is loosely used in society to describe a wide range of outcomes ranging from ways of playing, dancing, painting, and making music to exploring and experimenting in science and technology. There is evidence of creativity for as long as our history records extend back, ranging from ancient cave paintings and tools to contemporary music and new medical procedures. There have been many definitions of creativity in literature and academia (see Morgan 1953; Welsch 1981; Kampylis and Valtanen 2010; Elliot and Nakata 2013; Yin et al. 2021; Abraham 2022). For example, creativity can be defined as the forming of associative elements into new combinations which either meet requirements or are in some way useful. Conversely, creativity denotes a person’s capacity to produce new or original ideas, insights, inventions, or artistic products which are accepted by experts as being of scientific, aesthetic, social, or technical value. A definition with a modern twist on values is that creativity is imagination with responsibility (see Sae Ra Kung 2009).

We are familiar with the years of toil that can lead to a burst of new knowledge that sets a domain that others then occupy. The study of creativity reveals patterns to this type of creative burst. Creativity is often thought to exist on at least two levels, big C versus little c, eminent versus every day (Boden 2004). We can view creativity in terms of brilliance, personal creativity, paradigm or domain creativity, and forced or industrial creativity. This thinking on the significance of creative contribution was further extended by Kaufman and Beghetto (2009) to mini, little, pro, and big C creativity.

There are various historical periods associated with significant creative activity and development of our understanding (Sawyer 2011). In ancient Greece, patrons would support individual artists to creatively reflect the patron’s status. For example, the bankers’ guild of Florence commissioned a bronze statue of St. Matthew to decorate the church of Orsanmichele during the Renaissance. The Industrial Revolution can be characterized as leveraging the advantages of production and concentration of resource. Twentieth century contributions to creativity include increased understanding emerging from domains such as psychology and neuroscience. The second and third decades of the 21st century have seen great strides in automated and augmented creativity through data mining and artificial intelligence (AI). A modern-day analogy is the online platform Patreon which is a crowd-based service designed to support creators. A repeated insight from the study of creativity is the value of patronage, investment, and support for an activity. From individuals to institutions to nations and states, patronage pays for costly rigs, experiments, research and support teams. It enables innovation and the realization of an idea.

Research on creativity has addressed many areas of the topic, particularly in the years following JP Guildford’s call for attention to the topic in 1950 (Guilford 1950), with a focus on education, design, development, domain specificity, process, culture, traits, artificial intelligence, physiology, and neuroscience. In design and product development, creativity plays a vital role, especially during the early stages (Han et al. 2018a). Creativity is often considered a prerequisite for product innovation, ultimately leading to product success, which involves divergent and convergent thinking. The divergent phase of creativity is associated with the generation of many distinctive ideas, while the convergent phase is associated with the evaluation and refinement of ideas.

A number of approaches and tools have been developed for supporting idea generation as a part of the convergent phase of creativity, such as brainstorming (Osborn 1963), morphological analysis (Zwicky 1969), Method 6–3–5 (Rhorbach 1969), the Gallery Method (Pahl and Beitz 1996), SCAMPER (Eberle 1996), and C-Sketch (Shah et al. 2001). A recent study investigated the explorations of new problems in the divergent phase (Obieke et al. 2021). During the convergent phase, the consensual assessment technique (CAT), proposed by Amabile (1983), is often considered the gold standard for assessing creativity. Shah et al. (2003) proposed four metrics, namely, novelty, variety, quality, and quantity, for evaluating ideation effectiveness, while Fiorineschi et al. (Fiorineschi et al. 2022) refined the metrics to extend their applicability. Sarkar and Chakrabarti (2011) employed novelty and usefulness for measuring creativity, while Jagtap (2019) refined the novelty assessment method to better assess product novelty. Han et al. (2021) indicated that evaluating creativity should involve novelty, usefulness, and surprise. However, few studies to date have explored the divergent phase and the convergent phase of creativity as a whole. Therefore, a framework that considers both divergent and convergent thinking is needed to better support creativity.

Research within the groups associated with the principal author of this paper has resulted in 20 PhD theses of relevance to the broad topic of ideation. A common thread in the PhD studies concerned was a contribution toward an understanding of a fundamental facet of creativity with a view to enhancing creative practice, which lies in the field of creativity, design, innovation, and product development. These PhD studies covered a significant range of topics in creativity, including work on the systems model of creativity and creative engines (Wang 2013; Jiang 2015; Lee 2015), functional attributes (Tsai 2008; Michalakoudis 2019; Ekong 2014), creativity tools (Garvey 2016; Mi 2019), product creativity assessment (Hazeri 2019), individual differences (Yan 2017), combinational creativity (Han 2018), AI augmentations (Shi 2018; Chen 2020; DeWulf 2020), linkages among creativity, design, and innovation (Baxter 2017; Park 2018; Sikhwal 2020), innovative cultures (Grønneberg 2019; Garcia-Herrera 2020), and neurocognition (Wang 2021). The studies offered a comprehensive and extensive understanding of both divergent and convergent phases in creativity, whereas most other academic publications, such as journal and conference articles, only focused on specific topics or aspects of creativity.

The intent to enhance creativity is embedded in the multitude of good practice guidance on the topic and the many creativity tools. Therefore, on the basis of a review of the 20 PhD theses, along with the authors’ diverse experiences in design education (Childs 2019a), this research paper is aimed at proposing a new framework, consisting of a divergent phase for developing ideas and a convergent phase for refining ideas, to support users in selecting appropriate tools and approaches for innovative tasks in a creative environment. This new framework leverages commonly acclaimed approaches to creativity along with insights arising from the review, followed by experience in the use of the framework so far.

## 2. Creativity Process

In order to develop the new creativity framework, along with a review of the 20 PhD theses, this paper first reviews creativity process models to investigate the features and facets of creativity in this section. Creativity models that have been promoted for more than 10 years with the latest version having been evaluated by someone other than the promoters are reviewed. Human cognition is used to process information to achieve the creative process (Sweller 2009), and cognition is, therefore, also included in the review. Then, the paper presents some principal creativity tools and some of the common frameworks for creativity in the subsequent sections to form the basis of the newly proposed creativity framework.

Rhodes (1961) in his article “an analysis of creativity” portrayed four strands (person, process, press, and products), which, when combined, provide the functionality we refer to as creativity. These topics are expanded on in this section as they provide a basis for some of the development in the understanding of creativity.

Person in this context refers to our personality, intellect, temperament, traits, habits, attitudes, values, skills, and behavior. Process refers to the mental activities that occur during thinking. Wallas (1926) regarded creativity as comprising four stages:

(i) Preparation—this can involve observing, listening, asking, reading, collecting, and analyzing information.
(ii) Incubation—this involves both conscious and unconscious mental activity thinking about parts of an issue or opportunity, relationships between aspects and reasoning, sometimes with gaps of time between your conscious thinking and realizing you have formulated an idea worth taking forward.
(iii) Inspiration—the study of creativity suggests that worthwhile ideas sometimes arise when we are not deliberately addressing the topic and are associated with unconscious mental activity where the various pieces of information and memories come together in the form of a recognizable and worthwhile idea.
(iv) Verification—this involves the detailed work to convert an idea into its intended outcome, be it a physical or nonphysical form. Sadler-Smith (2015) added a fringe conscious subprocess that links incubation and illumination in this four-stage model and named it the intimation stage.

Press is used to describe the relationship between a person and their environment that influences and defines behaviors, development, and outcome. Rhodes used the term product to refer to the outcome of an idea being embodied in physical form. Today, we are used to services and systems that may not have a physical form; thus, the term product can be more broadly defined to refer to the final realization or manifestation of an idea, be it in the form of a physical artifact or nonphysical, perhaps in some form of digital media.

The use of the 4Ps, person, process, press, and product, to describe the various facets of creativity has been useful for many decades. Recent advances in neuroscience have suggested the addition of physiology, i.e., the way the brain works, as a further facet, as put forward by Abraham (2018). Sternberg and Karami (2022) further expanded consideration of creativity to purpose, press, person, problem, process, product, propulsion, and public, to provide a more complete treatment of the subject. A recent study by Girn et al. (2020) proposed a new framework to capture the complexity of dynamic shifts in neurocognitive states and the impact on creativity. Further research has been conducted to define neurocognitive constituents of creative thinking by utilizing neuroimaging during creative tasks to reveal the physiological impact of the prompts (Kenett et al. 2020).

Sustained attempts to describe and formalize the processes associated with creativity have been undertaken (Howard et al. 2008) to further develop our understanding of creativity. Some research has taken the view that creativity is a cognitive process (Miller 2014) related to distinct results (Abraham 2013), and that creativity can be achieved through creative idea generation and convergent thinking processes. The former is mainly based on divergent thinking and finding different creative solutions to problems, while the latter mainly concerns insights to problem solving (Benedek and Fink 2019; Lee and Ostwald 2022). Typically, creative ideas are generated on the basis of a combination of the two processes.

**Figure 1:**
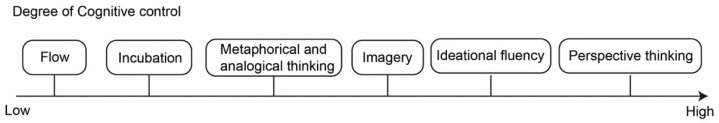
Schematic illustrating the basis for the Cognitive Processes Associated with Creativity (CPAC) scale.

The Cognitive Processes Associated with Creativity (CPAC) scale has a basis in the four-stage model originally developed by Wallas (1926) with phases of brainstorming, perspective taking, metaphorical and analogical thinking, incubation, imagery/sensory, and various subprocesses, as illustrated in Figure 1 (Miller 2014). Jacquet et al. (2020) reclassified the six subprocesses and provided further detailed explanations for each. Idea-generation (often referred to as brainstorming) is a process that attempts to generate as many potential responses or solutions as possible, regardless of the plausibility. Idea manipulation (perspective taking) is an intentional process that attempts to transform perception and perspective, allowing the individual to conceptualize or understand the situation in a different way. Metaphorical and analogical thinking is a process that produces a connection between the current problem and a similar or related situation, and then takes ideas from one context and applies them in a new setting. Incubation is a mental process where the person is engaged in other activities and generates ideas unconsciously. Imagery/sensory process is essential to internal visualization, which is a key element of the creative process. This process triggers creative ideas from the senses from different sensory modalities (such as auditory and tactile). Flow (Csikszentmihalyi [1996] 2019) is an almost automatic and highly focused state of consciousness process that occurs when an individual is engaged in intense work.

**Figure 2:**
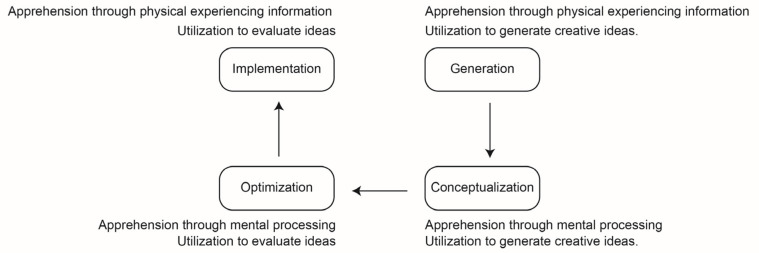
Combination of four different methods of gaining and using.

Basadur and Gelade (2005) promoted a differing four-stage cognitive process of creativity with stages of generating, conceptualizing, optimizing, and implementing. These stages are the combination of two factors: apprehension and utilization. Apprehension concerns the acquisition of understanding of knowledge through physical experience of information or mental processing. Utilization concerns applying understanding of knowledge to evaluate ideas or generate creative ideas (Figure 2). Basadur and Gelade’s model is a special cognitive process, combining the definition of problems and generating ideas into one stage, while, in other models, this is a single process where problems are defined and then ideas are generated.

In addition to the four-stage models, other principal models developed to describe creativity including the dual-process models, tripartite models, and tripartite-process models. Dual-process theory indicates that a cognitive thinking process involves two subprocesses: (i) autonomous idea generation; (ii) working memory-dependent idea revision, evaluation, and selection (Gabora et al. 2014).

The idea generation subprocess is considered to be an autonomous, rapid, nonconscious, and automatic process that enables associations through nature, gut reaction, and intuition (Leschziner and Brett 2019). Information residing in long-term memory is rapidly combined or associated with current-context information without effortful thinking and intervention. The idea revision, evaluation, and selection process is a rule-based, slow, controlled, effortful, conscious, and analytic process based on working memory (Leschziner and Brett 2019).

**Figure 3:**
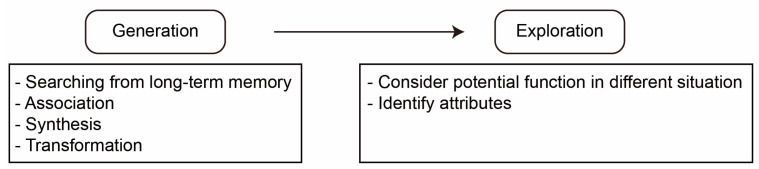
The Genoplore model.

Figure 3 depicts the Genoplore model, referring to the dual processes as generation and exploration (Finke et al. 1992). The generation process in the Genoplore model is a divergent thinking process which involves searching long-term memory, forming associations, synthesis, and transforming items. The exploration process is a convergent thinking process that involves considering potential functions in different situations.

**Figure 4:**
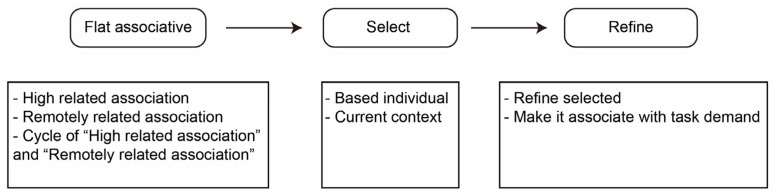
Gabora’s model.

Figure 4 shows Gabora’s (2010) model, which considers that, in the ideageneration process, people autonomously associate highly and remotely relative items from memory on the basis of stimulation (flat association), select ideas on the basis of individual characteristics and current conception, refine the selected idea, and form connections with task demands through associative and analytic thinking. Flat association is stimulated by current content.

**Figure 5:**
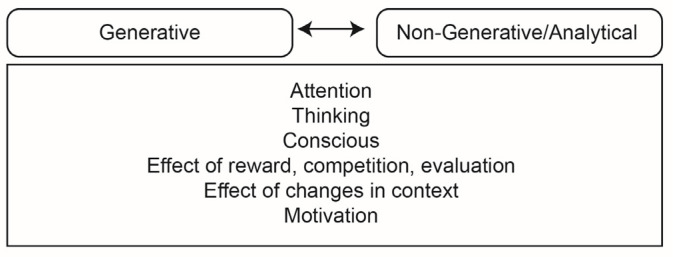
Howard-Jones’s model.

Figure 5 shows Howard-Jones’s (2002) model, which in essence is a summary of dual-process models. The model is based on association and divides the creative process into generative and nongenerative/analytical processes. The model highlights the importance of investigation, idea generation, self-evaluation, idea selection, design development, and outcome evaluation.

**Figure 6:**
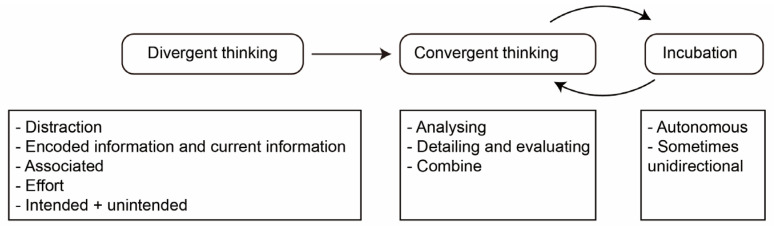
Structure of intellect model.

The structure of intellect model (Guilford 1956), shown in Figure 6, can be regarded as a dual-process model with divergent and convergent thinking processes. The divergent thinking process is an associated process where in distraction state, the encoded information is combined with information in current context (Gabora 2010). The convergent process, in contrast, is an analyzing process and is important in detailing and evaluating ideas. In this model, Guilford supports the view that the divergent thinking process happens before the convergent thinking process.

**Figure 7:**
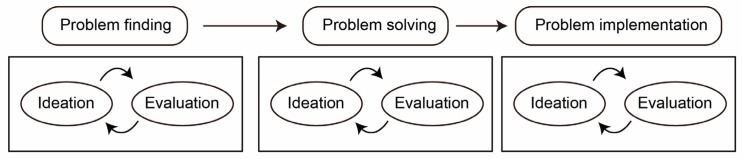
The ideation–evaluation cycle.

The ideation–evaluation cycle (Basadur et al. 1982) shown in Figure 7 divides the creative thinking process into three stages (problem finding, problem solving, and solution implementation). This model identifies that ideation and evaluation are involved in each stage to different degrees. This indicates that the dual process is repeated in different stages of creativity. However, this repeat theory is challenged. Self-report measurements, such as CPAC, have indicated that the cognitive process of creativity is based on “ideational fluency” where as many ideas as possible are generated, “metaphorical and analogical thinking” where ideas are applied from existing context to new content or combined, “perspective thinking” where people try to generate a specific solution for current problems, and “imagery” which is internal visualization (Miller 2014) (see Figure 1). This principle of CPAC indicates that creativity process is not a cyclic process. However, researchers for various of the creative process models promoted do not identify in which stage incubation and flow exist. According to the structure of intellect model, where incubation is promoted as part of cycles in the model, the incubation sub-process may repeat in the four stages. Whether the process is also cyclic is not certain because autonomous and conscious are contradictory to some degree.

**Figure 8:**
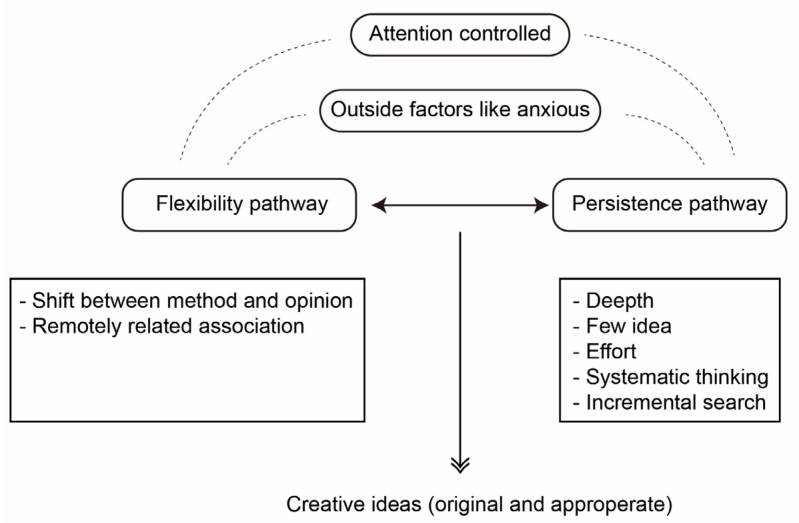
Dual pathway model schematic.

Nijstad et al.’s (2010) model, shown in Figure 8, presented the cognitive process in creativity on the basis of whether this creative process is conscious or unconscious, supporting the view that the creativity process is attention controlled and ideas are generated consciously.

**Figure 9:**
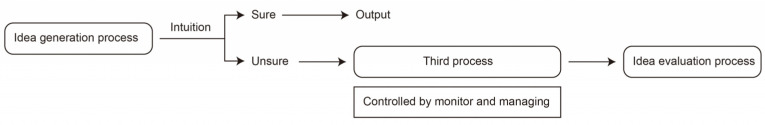
Tripartite-process model.

Despite being at the core for understanding the cognitive process of creativity, the dual-process theory has limitations. There is no clear boundary between idea generation (or divergent thinking process or flexibility pathway) and idea evaluation (or convergent thinking or persistence pathway). In the idea generation process, evaluating ideas is also needed to select the original ideas (Basadur et al. 1982; Campbell 1960). The two processes intervene with each other (Gabora et al. 2014). Researchers have acknowledged the existence of “intervention”. For example, Nijstad et al. (2010) explained this intervention as a shifting ability. The tripartite-process model, shown in Figure 9, separated out a new process from the dual-process theory. In the third process, how people deal with minds and behaviors is monitored and managed, influenced by individual personality and thinking preference, which is called cognitive style (Leschziner and Brett 2019). This tripartite-process model has a limitation as it indicates that the creativity process is an attention-controlled process similar to Nijstad et al.’s model. Therefore, sudden ideas generated from the creativity process are ignored.

Bhattacharya and Petsche’s (2005) model focused on which kind of memory was involved in the mental imagery idea generation process. Specifically, the model summarized the mental imagery idea generation process as three subprocesses: long-term memory to remember more patterns; reliance on visual memory or short-term memory to maintain; generation of possible graphic and active visual memory.

The models reviewed in this section can be divided into four principal types: (i) four-stage models, (ii) dual-process models, (ii) tripartite models, and (iv) tripartite-process models. The four-stage models and dual-process models play a core role in understanding the creativity process from cognitive aspects and connect creativity and consciousness. Some of these models are linear models which mainly include stages such as preparation, incubation, insight, verification, evaluation, and elaboration (Amabile 2012). Nearly all the models indicate that each process can be further detailed as more cognitive operations and cognitive factors (such as memory, association, and combination). Some common important features and facets in creativity mentioned in the diverse models include phasing, incubation, perspective, use of analogy and metaphor, association, exploration, stimulation, convergence and divergence, sequencing, attention, and evaluation. These facets of creativity are often implicitly or explicitly incorporated into process models for creativity and tools for enhancing creativity as considered in Section 3.

## 3. Creativity Tools

Creativity tools is a phrase often used to describe an approach offering enhanced generative outcomes. There are many creativity tools that are widely used, such as analogy, boundary shifting, various types of brainstorming, and checklists. Designers often employ these tools to facilitate the generation of creative ideas during early stages in design and product development. There are indeed hundreds of creativity tools available, most of which try to enhance (i) fluency—the quantity of responses, (ii) flexibility—ideas that are distinct from each other, or (iii) originality—the level of uniqueness of the ideas generated.

A creativity tool does not produce ideas. Instead, it can be used to assist in the generative process. Most tools can actually be used at any stage in a problem-solving process, but tend to mainly be focused on problem exploration, idea generation, and concept evaluation. Creativity tools generally function by (Childs and Fountain 2011) (i) ensuring that the problem can be understood in relatively simple terms so that this occupies only a fraction of your short-term memory, (ii) supplying cues to make the search of long-term memory more efficient, and (iii) providing cues to ensure refreshing of short-term memory and, thus, retention of key information.

There are a wide range of types of creativity tools including systematic or randomization approaches, the use of analogy and metaphor, and a series of brainstorming tools such as list, sticky note, alphabet, grid, and brainwriting. Examples of systematic tools are morphological analysis (Childs 2019b), TRIZ (the theory of inventive problem solving) including tools such as the contradiction matrix, the principle of inventions, Smart Little People, OTSM-TRIZ (General Theory of Powerful Thinking), and Size–Time–Cost Operator, along with tool selection guidelines (Moehrle 2005; Chechurin and Borgiani 2016; Fiorineschi et al. 2018), and SCAMPER (an acronym for the provocations substituting, combining, adapting, modifying, putting to another use, erasing, and rearranging). There is a growing number of computational creativity tools, such as Idea-Inspire 4.0 which can support analogical design employing a searchable biological systems knowledge base (Siddharth and Chakrabarti 2018), the Combinator which produces combinational textual and pictorial stimuli to promote users’ creative minds (Han et al. 2018b), and InnoGPS which retrieves design concepts from a multi-technology domain patent database (Luo et al. 2019). In recent years, AI techniques have been applied in computational creativity tools to better support users in creative tasks. For example, Shi et al. (2017) developed a design knowledge retrieval and association tool, B-Link, to support idea generation, which employs a large-scale ontology network constructed using unsupervised learning. Chen et al. (2019) proposed an AI-based approach using a generative adversarial network (GAN) to produce synthesized images of associated ideas in an ontology network to stimulate idea generation. Sarica et al. (2020, 2021) came up with a Technology Semantic Network (TechNet) developed using AI-based natural language processing (NLP) techniques, which could support a range of creative tasks, such as idea generation, idea evaluation, and knowledge management. The creativity diamond framework proposed in Section 4 prompts users to the availability of these tools at different stages in a creative challenge, and some of these tools are described in Section 5.

## 4. Creativity Diamond Framework

**Figure 10:**
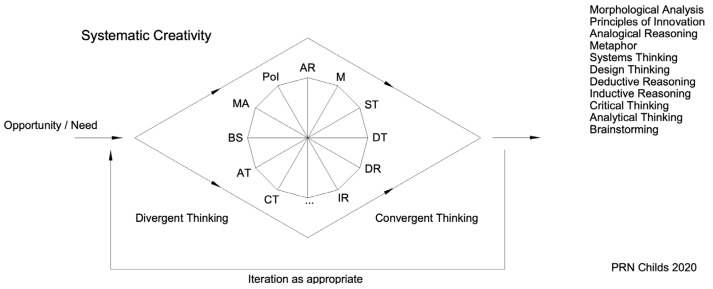
Creativity diamond framework.

The creativity diamond framework that is presented in this paper and illustrated in Figure 10 arose from a review of over 20 PhD theses supervised by the principal author over the last 20 years looking at aspects of creativity, design, innovation, and product development, along with consideration of previous frameworks and models. A doctoral thesis typically includes an overview of the relevant literature to define the extant knowledge base, as well as knowledge gaps and arising research questions. Consequently, in addition to identifying individual contributions from the research outputs, a given thesis also provides a readily accessible overview of research on creativity, despite some of the theses being up to 20 years old.

As identified in Section 2, substantive research on creativity has been undertaken, revealing the relevance of diverse facets including phasing, incubation, perspective, use of analogy and metaphor, association, exploration, stimulation, convergence and divergence, sequencing, attention, and evaluation. Many of these principles have been embodied in models and frameworks aimed at encouraging creativity. Examples of frameworks that have emerged in the domain of creativity include models for system creativity as embodied in the creativity engine (Childs and Fountain 2011), the six divergent and convergent phases in the creative problem-solving process (Treffinger et al. 2006), the readiness to recreation process model of Hasirci and Demirkan (2007), the five-stage creativity model of Amabile (1983, 1988) for individual or a small groups, use of the TASC wheel (see, for example, Faulkner 2008), creative design (Gero 1996), and the double diamond (Design Council 2019). Some common features of these include phases of divergence and convergence, as well as iteration.

Thoughts are built by our mind, i.e., how we think, feel, and choose, which generates electrochemical signals within our brain to build the thoughts from associated memories. Leaf (2018) suggests we are likely to have between 8000 and 10,000 thoughts a day, with each thought informed by our trillions of memories. Indeed, thoughts and ideas abound. One of the principles of brainstorming and some other approaches to creativity is that quantity of ideas breeds quality (see Osborn 1963; Childs 2019b). This can be a challenge to accept sometimes as we tend to cherish an idea that we have come up with during an initial phase of work. We may have invested some time in this and have a strong affinity to the idea. In looking at any other ideas, we may judge the newer ideas against the one originally generated and keep on returning to and working on the original idea. This is sometimes referred to as fixation (Jansson and Smith 1991). There may be merit in this original idea and, if so, it is worth keeping this idea available for subsequent consideration. History and research on creativity both indicate the value in exploring an idea space prior to deciding on which idea to select. There may be other areas worth considering. Someone else may have a better idea. In an industrial context, it may be worth exploring the idea space in detail to see what additional ideas exist and how a competitive company might respond to an innovation. Constructs for encouraging divergent thinking include consideration of what options and alternatives there are or might be.

A small group exploring and recording their ideas using sticky notes can produce many ideas over a few hours. Indeed, the use of a few types of brainstorming methods, in combination with ideas that emerge otherwise, can result in a few hundred ideas that need to be considered in order to identify which warrant further attention. The principles of brainstorming suggest suspension of judgement to promote an environment in which ideas can more readily emerge. Once a body of ideas has been established, a phase of selection and refinement of the ideas can take place. When considering the quality of an idea, specific aspects of the idea might become apparent and require attention to resolve an issue. This is a natural part of the convergent phase in idea generation. Convergent thinking involves focusing attention on the most appealing items in an extensive list of ideas. In evaluating which ideas to consider further and which to discard, one may choose to refer to the original brief to ensure that the idea is relevant and addresses the requirement.

The creativity diamond is a framework developed by incorporating fundamental principles to guide creativity. The framework embodies the principles of divergence, the generation of many ideas, such that, through selection and refinement, convergence can occur with selection of preferred ideas for further development. The creativity diamond framework is a guide that can be used in any domain or multidisciplinary setting to prompt which generative tools might be helpful.

A range of creativity tools and approaches to thinking creatively are presented in the creativity diamond, including various types of brainstorming, morphological analysis (see Garvey 2016; Childs and Garvey 2015), the principles of invention from TRIZ—the theory of inventive problem solving (see Gadd 2011; Tsai 2008), analogical reasoning (Han 2018; Han et al. 2018a; Mi 2019), use of metaphor (Mi 2019), systems thinking, design thinking, deductive and inductive reasoning (Park 2018; Park et al. 2020), and critical and analytical thinking.

The supporting information for the creativity diamond helps guide the selection of which creativity tool to use for a given type of person and when. For example, one might choose to use sticky note brainstorming, morphological analysis, aspects of design thinking, and use of metaphor during a divergent process to help generate ideas for a given opportunity or problem. Then, during a convergent phase of idea selection and refinement one might make further use of one of the types of brainstorming, critical thinking, the principles of invention, and analogical reasoning.

The creativity diamond presents activity as commencing in response to a need or opportunity. It may be that an idea to explore an area just arises or comes in response to a defined need as part of your work. The convergent activity may result in the selection of a preferred idea along with some refinement and embodiment of this idea. It is also possible that the convergent phase identifies a few or several ideas worth further consideration. The creativity diamond incorporates an iterative loop such that further phases of divergence and convergence can occur with these ideas in order to further develop the ideas, solve problems, and refine aspects as needed. Iteration is also implicit to many of the creativity tools and approaches to thinking which may result in cycling around an issue to address it within both the divergent and the convergent phases.

## 5. Types of Thinking and Tools

The creativity diamond presents a divergent phase to encourage the generation of many ideas for consideration and a convergent phase for the selection of preferred ideas and their refinement. Some of the many approaches to creative thinking are presented by means of the central graphic. These can be selected on the basis of preference, timeframe, a desire to use different types of approaches to creativity, and application. Table 1 provides a brief introduction and guide to the various approaches presented.

## 6. Use and Justification

Much of the experience in the use of creativity tools for the groups concerned with the authors of this paper arises from experiences in the multidisciplinary group project run across all undergraduate degrees at Imperial College London, Innovation Design Engineering (IDE) program, a double master’s program run by the Royal College of Art and Imperial College London (see Childs and Pennington 2015), its “sibling” program Global Innovation Design (GID) (see Stevens et al. 2015), and the Design Engineering mEng in the Dyson School of Design Engineering at Imperial College London (see Childs 2019a). In addition, experiences have arisen from interactions with other universities, in industry, and in the Creative Thinking Tools for Success MOOC (Massive Online Open Course) on the Coursera and EdX platforms, in which over 300,000 people have engaged to date. In the case of the Coursera Creative Thinking Tools for Success MOOC, at the point of analysis, 238,749 people had enrolled with 3479 ratings at an average of 4.7/5, 97% positive endorsements, and 1046 reviews. The text responses were diverse and with 97% positive endorsements, overwhelmingly favorable, highlighting the overview of and access to the diverse creativity tools and thinking approaches.

Diverse creativity tools have been introduced to generations of students on the IDE, GID, and Design Engineering MEng programs to supplement innate approaches. The culture for the programs concerned is often characterized by student choice. Despite definition of learning outcomes, each student’s pathway is unique as a result of definition of their own solo and group projects and electives. It is noted that the selective nature of the programs means that the population of students will not be representative of society, with high levels of qualification attainment and the freedom to select an area of domain preference within which to study. A typical introduction of a particular tool or approach to thinking will involve exposure to the approach, exploration of the fundamental principles of operation along with use of the tool for some examples or application. Students are subsequently free to use the approach at their discretion. The nature of design portfolios and narrative means that it is sometimes possible to identify use of a tool or approach through, for example, inclusion of a snapshot of hundreds of ideas on sticky notes, a matrix showing systematic exploration of an idea space, or the student’s reflective narrative.

Students tend to have multiple exposures to the diverse forms of brainstorming such as sticky note, list, grid, alphabet, and brainwriting across different modules in their studies. Circle brainstorming, with its origin at Imperial, tends to be used for specific modules such as the Billion Dollar Question module where repeated pitching of an idea and its rapid revision based on consultant input prior to further pitching is compatible with the intense nature of the module. Systematic tools such as morphological analysis, TRIZ, and SCAMPER are introduced in specific sessions. Biomimicry, in a similar way to brainstorming, is widely introduced across different modules. Subjects such as the use of metaphor and analogy have historically tended to be introduced in passing, allowing a student to develop their skills and abilities in the subject through application across diverse projects. The use of AI to augment creativity and AI tools is usually associated with seminar presentation and students are subsequently given access to the tools concerned. Design thinking and systems thinking are introduced across a broad range of modules and projects ranging from Context in Design Engineering and throughout the first year of the Innovation Design Engineering and Global Innovation Design MScs. Specific topics on thinking such as critical and analytical thinking are introduced through principles and examples.

**Figure 11:**
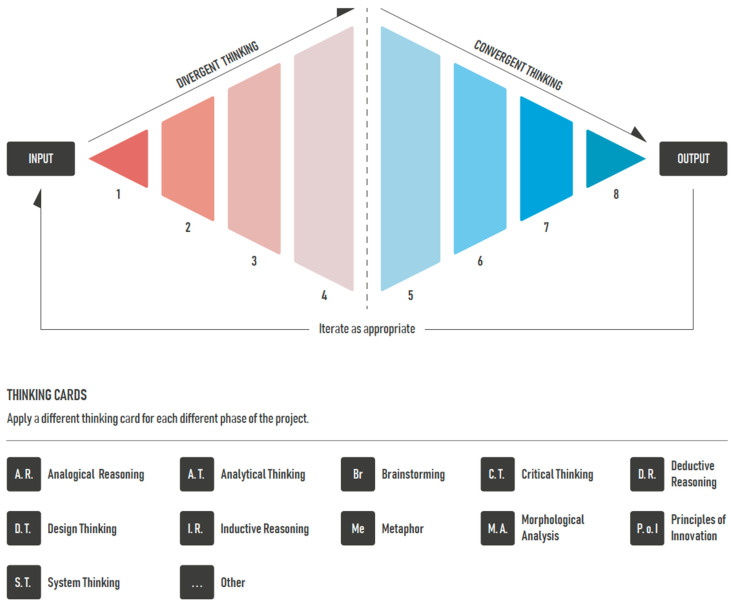
Creativity diamond graphic.

**Figure 12:**
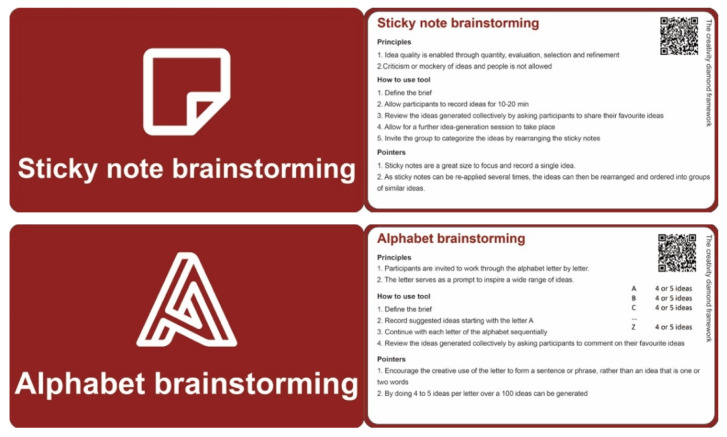

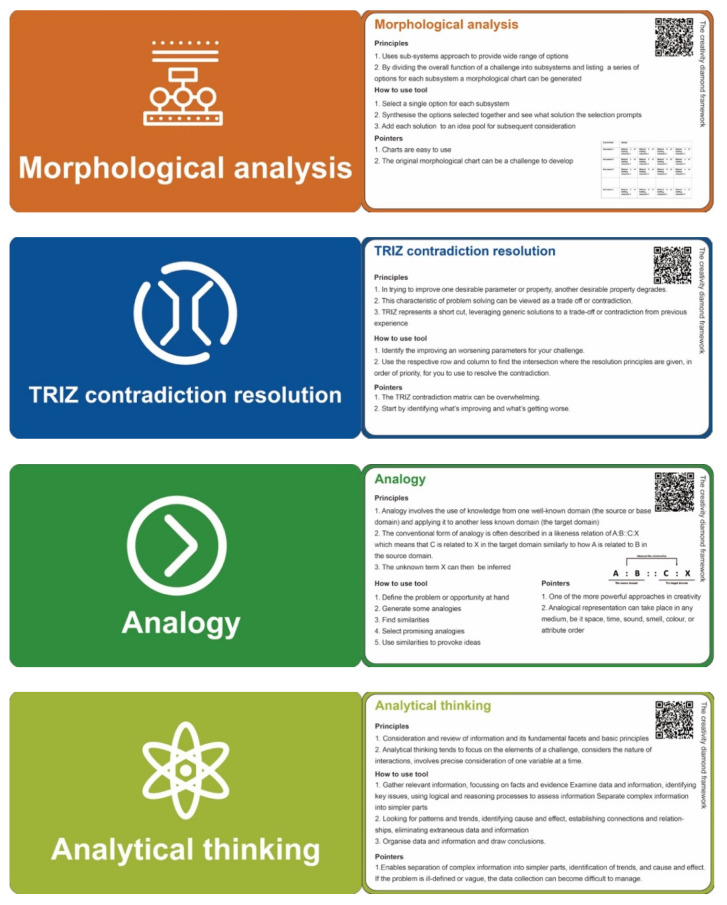
Examples of resource cards for some of the creativity tools.

The wide range of tools and approaches available, particularly the approaches to thinking, can be overwhelming to a student group with recourse to familiar approaches such as sticky-note brainstorming and biomimicry common in the past. The “single graphic” in various forms (see, for example, Figure 11) for the presentation of the different approaches to creativity, along with encouragement to use more than one approach, has challenged this culture with students and groups of students exploring the use of a few approaches within both divergent and convergent phases of activity. The use of attribute tables such as that presented in Table 2, along with resource cards and associated links (Figure 12), has also been quickly adopted, allowing individual preferences, application, and time factors to be quickly considered in the selection of an approach. For example, “list brainstorming” is a low-difficulty tool that can be used either solo or in a group for less than 1 h. It is a tool suitable for extroversion personality traits aimed at producing little c outputs. The examples of resource cards presented in Figure 12 are part of a set of cards representing each of the creativity tools and types of thinking, serving as a prompt for an individual or team for the tool concerned.

## 7. Conclusions

The creativity diamond is a framework developed to aid users in the selection of an approach to augment creativity and creative thinking in divergent and convergent phases of a challenge or opportunity, particularly in design and product development. The framework has arisen from a review of over 20 PhD theses on creativity, design, innovation, and product development, which in turn build on their respective domain literature reviews. The framework promotes several forms of brainstorming, a series of systematic approaches to creativity, and several thinking and reasoning approaches. To accompany the framework, several resources have been developed, including upskilling materials in each of the approaches, summary information, and selection tools to aid identification of an approach at a given stage. The framework has been introduced across several degree programs at Imperial and other universities, as well as in industry, through a massive online open course. The creativity diamond framework is proposed as a guide that can be used in any domain or multidisciplinary setting to prompt which generative tools might be helpful to aid creativity, ultimately leading to innovation success.

## Figures and Tables

**Table 1:** Short description and merits/limitations of various creativity tools and approaches to thinking.

Tool/Approach	Description	Merits	Limitations
Brainstorming	A set of approaches based on the principle that quantity of ideas breeds quality through subsequent selection and refinement.	Widely used approach that can help generate a range of ideas and alternative perspectives.	Can support generating a large amount of ideas but requires multistage selection and refinement. Relies on several participants to leverage benefits.
Morphological analysis	By dividing a challenge into subsystems and providing options for each subsystem, selections can be made to explore a range of arising combinations.	Enables alternative ideas to be formulated from a set of options.	Can be very time-consuming; lack of methods to assess whether the ideas developed effectively address the brief.
Principles of invention	A list of 40 fundamental approaches commonly found in patents for resolving a challenge	The 40 principles provide a comprehensive set of approaches used in patents to resolve challenges across a very wide set of, if not all, domains.	Limited application to physical problems. However, needs to be abstracted to apply to nonphysical problem briefs, such as service innovation.
Analogical reasoning	Use of a similarity between a source and target to support an assertion	Enables transfer knowledge from one area to another.	Requires preparation to select examples that can be used for analogical reasoning.
Metaphor	Use of association between a commonplace idea with something that is unfamiliar to provoke understanding and ideas	By combining elements that have sparse or no obvious logical connection, metaphors enable the mind to be stimulated by images, ideas, and concepts, thereby exploring ideas that are distinct from logical relations.	Randomness in metaphor choice can lead to unrelated ideation with little benefit to address original brief, despite being a powerful tool to motivate and create mindshift to address a brief.
Systems thinking	A set of analytical approaches used to model interrelated, interdependent, or interacting elements forming collective entities in order to provide predictions and enable control.	Enables understanding of the behaviour of collective entities, in order to provide predictions and control.	Supports clarifying a problem or situation, but barriers to developing provocative and breakthrough ideas.
Design thinking	Emulating some of the approaches that designers have traditionally used to realize their ideas, such as a user-centred focus, experimentation, prototyping, testing, and toleration of ambiguities until sufficient information is available.	Promotes consideration of the voice of the customer.	This technique often relies on user insights, which are time-consuming to obtain.
Deductive reasoning	Starts with a hypothesis and then examines possibilities and data to reach a conclusion.	Powerful for information-rich applications when significant information processing resources are available.	This technique relies heavily upon the initial premises being correct. This can prove especially difficult in context with many unpredictable variables with a lack of constants or controls. If used in a team setting, it can lead to frustration of participants
Inductive reasoning	Broad conclusions are inferred from a specific case and used to provide the basis for a generalization that the pattern of behavior is applicable to a much wider set of situations.	Enables insight from sparse data.	Conclusions drawn can be difficult to prove and have limited significance. Since this approach relies on observation, there can be limitations if the observations are incorrect or incomplete. Incomplete observations can lead to flawed conclusions.
Critical thinking	An organized and rational approach to enable evaluation of information and its interpretation.	Steps such as identifying the problem, data gathering, data evaluation, identifying any assumptions and bias, establishing the significance of information, making a decision, and conclusion can be readily followed.	It can be time-consuming to gather facts, sort facts from fiction, and consider the quality of the sources of information.
Analytical thinking	Consideration and review of information and its fundamental facets and basic principles	Enables separation of complex information into simpler parts, identification of trends, and cause and effect.	Can be time-consuming and challenging to make decisions. In a team setting this approach, can lead to frustration and induce the feeling of indecisiveness. This approach depends on the skill of the data analyst and the quality of data sources. Often, this approach sets out with a defined problem. If the problem is ill-defined or vague, the data collection can become difficult to manage.
…	Other tools and approaches that can be added to the diamond.	Allows for additional methods to be added and considered.	

**Table 2:** Creativity approach attributes. X = indicative; times are approximate; levels are indicative; L = low; M = medium; H = high; U = unspecified; h = hour.

	Solo	Group	Extroversion Aligned	Introversion Aligned	Small c Aligned	Big C Aligned	Time	Difficulty Level	AI Version Readily Available
List brainstorming	x	x	x		x		<1 h	L	x
Sticky-note brainstorming	x	x		x	x		<1 h	L	
Grid brainstorming		x		x	x		<2 h	L	
Alphabet brainstorming	x	x	x	x	x		<2 h	L	
Brainwriting		x		x	x		<2 h	L	
Circle brainstorming		x	x		x		<2 h	L	
Morphological analysis	x	x		x	x		<2 h	L	x
TRIZ contradiction resolution	x	x		x	x		<2 h	M	
TRIZ smart little people	x	x	x	x	x		<1 h	L	
SCAMPER	x	x	x	x	x		<1 h	L	
Analogical reasoning	x	x		x	x	x	U	H	
Analogy	x	x		x	x	x	U	H	
Biomimicry	x	x		x	x	x	U	M	
Metaphor	x	x		x	x	x	U	H	
Systems thinking	x	x		x	x		U	M	
Design thinking	x	x	x	x	x		U	M	
Deductive reasoning	x	x		x	x		U	M	
Inductive reasoning	x	x		x	x		U	M	
Critical thinking	x	x	x	x	x		<2 h	L	
Analytical thinking	x	x	x	x	x		<2 h	L	

## Data Availability

Not applicable.

## Nomenclatures

The following abbreviations are used in the article and associated graphics:

AR	analogical reasoning
AT	analytical thinking
BS	brainstorming
c, C	creativity
CPAC	Cognitive Processes Associated with Creativity scale
CT	critical thinking
DR	deductive reasoning
DT	design thinking
GID	Global Innovation Design
IDE	Innovation Design Engineering
IR	inductive reasoning
M	metaphor
MA	morphological analysis
PoI	principles of invention
ST	systems thinking
TRIZ	Theory of Inventive Problem Solving
